# A stoichiometric perspective of the effect of herbivore dung on ecosystem functioning

**DOI:** 10.1002/ece3.3666

**Published:** 2017-12-15

**Authors:** Judith Sitters, Harry Olde Venterink

**Affiliations:** ^1^ Ecology and Biodiversity Department Biology Vrije Universiteit Brussel Brussels Belgium; ^2^ Mpala Research Centre Nanyuki Kenya; ^3^ Departments of Aquatic and Terrestrial Ecology Netherlands Institute of Ecology (NIOO‐KNAW) Wageningen The Netherlands

**Keywords:** feces, grazing, N:P ratio, nitrogen, phosphorus, plant competition, stoichiometry, tree–grass balance

## Abstract

Ungulate herbivores play a prominent role in maintaining the tree–grass balance in African savannas. Their top‐down role through selective feeding on either trees or grasses is well studied, but their bottom‐up role through deposition of nutrients in dung and urine has been overlooked. Here, we propose a novel concept of savanna ecosystem functioning in which the balance between trees and grasses is maintained through stoichiometric differences in dung of herbivores that feed on them. We describe a framework in which N_2_‐fixing trees and grasses, as well as ungulate browsing and grazing herbivores, occupy opposite positions in an interconnected cycle of processes. The framework makes the testable assumption that the differences in dung N:P ratio among browsers and grazers are large enough to influence competitive interactions between N_2_‐fixing trees and grasses. Other key elements of our concept are supported with field data from a Kenyan savanna.

Savanna ecosystems are characterized by a mixture of grasses and trees (Scholes & Archer, 1997). In African savannas, the grasses are mainly of the C_4_‐photosynthetic pathway and many of the trees, such as the abundant *Acacia* (now sometimes called *Vachellia* and *Senegalia*), can symbiotically fix atmospheric nitrogen (N). African savannas also support the highest abundance and diversity of extant ungulate herbivores (Du Toit & Cumming, 1999). By feeding selectively on either trees (browsers) or grasses (grazers), these herbivores exert a direct, top‐down influence upon the balance between woody and herbaceous components of savanna vegetation. However, herbivores can also influence the structure of vegetation indirectly, for example, by altering fire frequencies or the competitive interactions between grasses and trees (Augustine & McNaughton, 2004; Du Toit & Cumming, 1999; Holdo, Holt, Coughenour, & Ritchie, 2007; van Langevelde et al., 2003; Riginos & Grace, 2008; Riginos & Young, 2007; Sankaran, Ratnam, & Hanan, 2008; Scholes & Archer, 1997; Staver, Bond, Stock, van Rensburg, & Waldram, 2009).

In addition to these top‐down processes, herbivores also affect vegetation by producing dung and urine, which returns nutrients such as N and phosphorus (P) in a more rapidly available form than through litter decomposition (Bardgett & Wardle, 2003). Indeed, the magnitude of nutrient returns in excreta can be comparable to inputs through wet atmospheric deposition and leaf litter decomposition (Augustine, McNaughton, & Frank, 2003; Cech, Olde Venterink, & Edwards, 2010; Fornara & Du Toit, 2008; Sitters, Edwards, & Olde Venterink, 2013; Sitters, Maechler, Edwards, Suter, & Olde Venterink, 2014; van der Waal et al., 2011). However, little attention has been paid to the fact that different savanna herbivores produce dung containing widely different concentrations of N and P and that these nutrients are released at different rates (Sitters et al., 2014). As these nutrients are often limiting in savanna vegetation (Augustine et al., 2003; Cech, Kuster, Edwards, & Olde Venterink, 2008), such variation in the supply ratios of N to P can influence plant species composition (Olde Venterink & Guesewell, 2010), and we might expect tree–grass interactions to be affected by the types of dung returned to the ecosystem. It appears, however, that this possibility has largely been overlooked.

Here, we propose a stoichiometric concept of savanna ecosystem functioning in which N_2_‐fixing trees and grasses, as well as ungulate browsers and grazers (either wild or domestic), occupy opposite positions in an interconnected cycle of processes (Figure 1a): N_2_‐fixing trees produce relatively N‐rich leaves with a high N:P ratio (Figure 1b), and browsers consuming these leaves, for example, giraffe, produce dung with relatively high N:P ratios (Figure 1c). In contrast, C_4_‐grasses have relatively N‐poor leaves with low N:P ratios (Figure 1b), and grazers consuming this herbage, for example, zebra, produce dung with low N:P ratios (Figure 1c). We make the testable assumption that the differences in dung N:P ratio between browsers and grazers, in combination with differences in dung quantity, are large enough to influence competitive interactions between N_2_‐fixing trees and grasses, particularly when the trees are still seedlings (Figure 1a). Thus, the pathway of nutrient return through herbivore dung could play an important role in maintaining the tree–grass balance in savannas.

**Figure 1:**
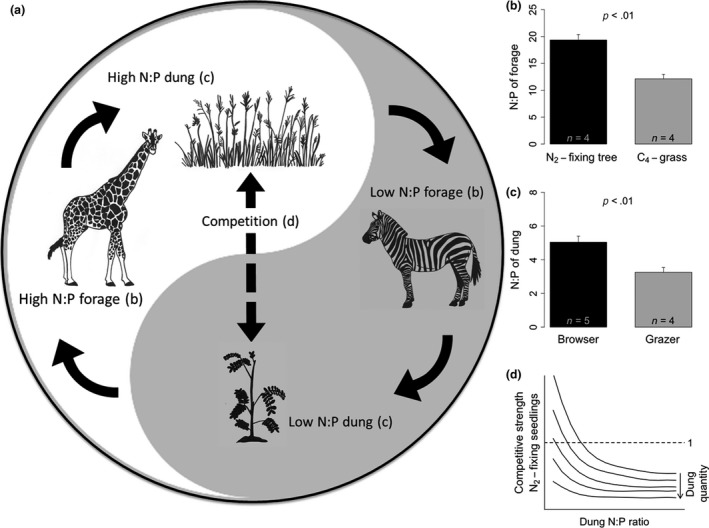
Conceptual framework of African savanna where N_2_‐fixing trees and grasses, as well as ungulate browsers and grazers, occupy opposite positions in an interconnected cycle of processes (a). Forage (b) and dung samples (c) were collected in a semiarid savanna at the Mpala Research Centre (36°52′E, 0°17′N) in Kenya. Leaves of six individual trees of the N_2_‐fixing species *Acacia (Senegalia) brevispica*,* A. mellifera*,* Acacia* (*Vachellia) drepanolobium,* and *A. gerrardii* were collected, together with leaves of up to 20 individuals of the dominant C_4_‐grass species *Brachiaria lachnantha*,* Pennisetum mezianum*,* P. stramineum*, and *Themeda triandra* in January 2016. Additionally, fresh dung of the browser species Guenther's dikdik (*n* = 6), eland (*n* = 3), giraffe (*n* = 5), greater kudu (*n* = 3) and Thomson's gazelle (*n* = 6), and the grazer species African buffalo (*n* = 5), Burchell's zebra (*n* = 6), Grevy's zebra (*n* = 2), and common warthog (*n* = 3) were collected. Forage and dung samples were dried, ground, and analyzed for total N and P concentrations. Classification of a herbivore species as a browser or grazer was based on dung δ^13^C values, with browsers ranging from −28 to −25 and grazers from −16 to −13, according to (Codron & Codron, 2009; Codron et al., 2007). Comparisons between N_2_‐fixing trees and C_4_‐grasses, and between browser and grazer dung, were performed with balanced ANOVA and unbalanced ANOVA, respectively. Panel (d) shows hypothesized effects of dung N:P ratio and dung quantity on the competitive strength of N_2_‐fixing tree seedlings; that is, biomass when growing in a mixture with C_4_‐grasses relative to biomass in the absence of the competing grasses (values >1 indicate that tree seedlings are superior competitors and values <1 that grasses are superior)

We hypothesize that a low quantity of dung with a low N:P ratio will create N‐limited conditions and stimulate symbiotic N_2_‐fixation by the tree seedlings (Vitousek et al., 2002), giving them a competitive advantage over grasses (Figure 1d). However, with an increase in dung quantity, the absolute amounts of available N and P increase regardless of the dung N:P ratio; under these conditions, we would expect less N_2_‐fixation, as this process is constrained by high soil N availability (Pons, Perreijn, van Kessel, & Werger, 2007). Under these conditions of high soil N and P availabilities, grasses are likely to become dominant as they generally have higher plant N and P use efficiencies than N_2_‐fixing tree seedlings (Lambers, Raven, Shaver, & Smith, 2008; Sage & Pearcy, 1987; Tjoelker, Craine, Wedin, Reich, & Tilman, 2005). Dung with a high N:P ratio, regardless of dung quantity, will also stimulate the growth of grasses, because the ability of symbiotic N_2_‐fixation is no longer decisive under these more P‐limited conditions, while P use efficiency is.

This concept, illustrated in Figure 1, provides an innovative perspective not only on the functioning of savanna ecosystems, but on the functioning of grazing ecosystems around the globe, because it links the following “observations” that have not been connected before: (1) Herbivores not only influence vegetation composition through (selective) plant consumption, but also through their dung and urine that serve as natural fertilizers, (2) dung from herbivore species varies considerably in C:N:P stoichiometry because of variation in quality of their diet, and (3) N:P stoichiometry of nutrient supply influences plant species composition.

We have supported our concept with data collected in a Kenyan savanna (Figure 1). These data are consistent with N:P ratios measured in forage (Augustine et al., 2003; Cech et al., 2008; Craine, Morrow, & Stock, 2008; Ludwig, de Kroon, Prins, & Berendse, 2001; Sitters et al., 2013) and dung in other African savannas (de Iongh et al., 2011; Sitters et al., 2014; van der Waal et al., 2011), although no other study has examined all the elements of Figure 1. Obviously, the impact of N and P availabilities and their stoichiometry on plant species competition in our concept still needs verification, and this article is meant as an encouragement to obtain these data. Previous research on the constraint of woody recruitment in abandoned livestock areas, which exhibit much higher soil N and P availabilities than the surrounding savanna landscape, is in accordance with our concept (Augustine, 2003; van der Waal et al., 2011), as is the prediction that N‐poor environments are more prone to woody encroachment (Kraaij & Ward, 2006). Moreover, at least one study has shown lower competitive suppression of N_2_‐fixing seedlings by grasses under low soil N:P ratios (Cramer, van Cauter, & Bond, 2010). We also have experimental evidence that variation in mineral N:P supply is a potential driver of competitive exclusion among dominant plant species (Olde Venterink & Guesewell, 2010) and that dung of different European herbivores impacted the composition and diversity of an experimental plant community (Valdés Correcher, Sitters and Olde Venterink, unpublished data).

It is important to note that the supply ratio of N:P might only have an effect on tree–grass interactions in savanna when water availability is not limiting plant growth, because drought might override the N:P effect on tree–grass competition (Scholes & Archer, 1997). Also, the functional variability in life stages of grasses and particularly trees may influence the processes presented in Figure 1. For instance, seedlings of N_2_‐fixing species will be more affected by competition from C_4_‐grasses than mature trees under “high absolute levels of N and P” and/or “high N:P conditions,” because roots of adult trees may explore deeper soil P pools than the seedlings or the grasses (Holdo & Nippert, 2015).

The concept described here may have a stabilizing effect on the long‐term functioning of savanna ecosystems. Variation in herbivore dung stoichiometry can stimulate a patchy landscape, whereby a patch occupied by trees will subsequently become a grassland patch, and vice versa. This would imply that soil conditions remain spatially uniform in the long term; that is, there is no tendency for tree‐dominated areas to become progressively enriched with N or grass‐dominated areas to become depleted, as previously described (Cech et al., 2008).

## CONFLICT OF INTEREST

None declared.

## References

[ece33666-bib-0001] Augustine, D. J. (2003). Long‐term, livestock‐mediated redistribution of nitrogen and phosphorus in an East African savanna. Journal of Applied Ecology, 40, 137–149. https://doi.org/10.1046/j.1365-2664.2003.00778.x

[ece33666-bib-0002] Augustine, D. J. , & McNaughton, S. J. (2004). Regulation of shrub dynamics by native browsing ungulates on East African rangeland. Journal of Applied Ecology, 41, 45–58. https://doi.org/10.1111/j.1365-2664.2004.00864.x

[ece33666-bib-0003] Augustine, D. J. , McNaughton, S. J. , & Frank, D. A. (2003). Feedbacks between soil nutrients and large herbivores in a managed savanna ecosystem. Ecological Applications, 13, 1325–1337. https://doi.org/10.1890/02-5283

[ece33666-bib-0004] Bardgett, R. D. , & Wardle, D. A. (2003). Herbivore‐mediated linkages between aboveground and belowground communities. Ecology, 84, 2258–2268. https://doi.org/10.1890/02-0274

[ece33666-bib-0005] Cech, P. G. , Kuster, T. , Edwards, P. J. , & Olde Venterink, H. (2008). Effects of herbivory, fire and N2‐fixation on nutrient limitation in a humid African savanna. Ecosystems, 11, 991–1004. https://doi.org/10.1007/s10021-008-9175-7

[ece33666-bib-0006] Cech, P. G. , Olde Venterink, H. , & Edwards, P. J. (2010). N and P cycling in Tanzanian humid savanna: Influence of herbivores, fire, and N2‐fixation. Ecosystems, 13, 1079–1096. https://doi.org/10.1007/s10021-010-9375-9

[ece33666-bib-0007] Codron, D. , & Codron, J. (2009). Reliability of delta C‐13 and delta N‐15 in faeces for reconstructing savanna herbivore diet. Mammalian Biology, 74, 36–48. https://doi.org/10.1016/j.mambio.2007.12.005

[ece33666-bib-0008] Codron, D. , Codron, J. , Lee‐Thorp, J. A. , Sponheimer, M. , de Ruiter, D. , Sealy, J. , … Fourie, N. (2007). Diets of savanna ungulates from stable carbon isotope composition of faeces. Journal of Zoology, 273, 21–29. https://doi.org/10.1111/j.1469-7998.2007.00292.x

[ece33666-bib-0009] Craine, J. M. , Morrow, C. , & Stock, W. D. (2008). Nutrient concentration ratios and co‐limitation in South African grasslands. New Phytologist, 179, 829–836. https://doi.org/10.1111/j.1469-8137.2008.02513.x 18537887 10.1111/j.1469-8137.2008.02513.x

[ece33666-bib-0010] Cramer, M. D. , van Cauter, A. , & Bond, W. J. (2010). Growth of N2‐fixing African savanna Acacia species is constrained by below‐ground competition with grass. Journal of Ecology, 98, 156–167. https://doi.org/10.1111/j.1365-2745.2009.01594.x

[ece33666-bib-0011] de Iongh, H. H. , de Jong, C. B. , van Goethem, J. , Klop, E. , Brunsting, A. M. H. , Loth, P. E. , & Prins, H. H. T. (2011). Resource partitioning among African savanna herbivores in North Cameroon: The importance of diet composition, food quality and body mass. Journal of Tropical Ecology, 27, 503–513. https://doi.org/10.1017/S0266467411000307

[ece33666-bib-0012] Du Toit, J. T. , & Cumming, D. H. M. (1999). Functional significance of ungulate diversity in African savannas and the ecological implications of the spread of pastoralism. Biodiversity and Conservation, 8, 1643–1661. https://doi.org/10.1023/A:1008959721342

[ece33666-bib-0013] Fornara, D. A. , & Du Toit, J. T. (2008). Browsing‐induced effects on leaf litter quality and decomposition in a southern african savanna. Ecosystems, 11, 238–249. https://doi.org/10.1007/s10021-007-9119-7

[ece33666-bib-0014] Holdo, R. M. , Holt, R. D. , Coughenour, M. B. , & Ritchie, M. E. (2007). Plant productivity and soil nitrogen as a function of grazing, migration and fire in an African savanna. Journal of Ecology, 95, 115–128. https://doi.org/10.1111/j.1365-2745.2006.01192.x

[ece33666-bib-0015] Holdo, R. M. , & Nippert, J. B. (2015). Transpiration dynamics support resource partitioning in African savanna trees and grasses. Ecology, 96, 1466–1472. https://doi.org/10.1890/14-1986.1

[ece33666-bib-0016] Kraaij, T. , & Ward, D. (2006). Effects of rain, nitrogen, fire and grazing on tree recruitment and early survival in bush‐encroached savanna, South Africa. Plant Ecology, 186, 235–246. https://doi.org/10.1007/s11258-006-9125-4

[ece33666-bib-0017] Lambers, H. , Raven, J. A. , Shaver, G. R. , & Smith, S. E. (2008). Plant nutrient‐acquisition strategies change with soil age. Trends in Ecology & Evolution, 23, 95–103. https://doi.org/10.1016/j.tree.2007.10.008 18191280 10.1016/j.tree.2007.10.008

[ece33666-bib-0018] Ludwig, F. , de Kroon, H. , Prins, H. H. T. , & Berendse, F. (2001). Effects of nutrients and shade on tree‐grass interactions in an East African savanna. Journal of Vegetation Science, 12, 579–588. https://doi.org/10.2307/3237009

[ece33666-bib-0019] Olde Venterink, H. , & Guesewell, S. (2010). Competitive interactions between two meadow grasses under nitrogen and phosphorus limitation. Functional Ecology, 24, 877–886. https://doi.org/10.1111/j.1365-2435.2010.01692.x

[ece33666-bib-0020] Pons, T. L. , Perreijn, K. , van Kessel, C. , & Werger, M. J. A. (2007). Symbiotic nitrogen fixation in a tropical rainforest: 15N natural abundance measurements supported by experimental isotopic enrichment. New Phytologist, 173, 154–167. https://doi.org/10.1111/j.1469-8137.2006.01895.x 10.1111/j.1469-8137.2006.01895.x 17176402

[ece33666-bib-0021] Riginos, C. , & Grace, J. B. (2008). Savanna tree density, herbivores, and the herbaceous community: Bottom‐up vs. top‐down effects. Ecology, 89, 2228–2238. https://doi.org/10.1890/07-1250.1 18724733 10.1890/07-1250.1

[ece33666-bib-0022] Riginos, C. , & Young, T. P. (2007). Positive and negative effects of grass, cattle, and wild herbivores on Acacia saplings in an East African savanna. Oecologia, 153, 985–995. https://doi.org/10.1007/s00442-007-0799-7 17661089 10.1007/s00442-007-0799-7

[ece33666-bib-0023] Sage, R. F. , & Pearcy, R. W. (1987). The nitrogen use efficiency of C3 and C4 plants. 2. Leaf nitrogen effects on the gas‐exchange characteristics of *Chenopodium album* (L.) and *Amaranthus retroflexus* (L.). Plant Physiology, 84, 959–963. https://doi.org/10.1104/pp.84.3.959 16665551 10.1104/pp.84.3.959 PMC1056702

[ece33666-bib-0024] Sankaran, M. , Ratnam, J. , & Hanan, N. (2008). Woody cover in African savannas: The role of resources, fire and herbivory. Global Ecology and Biogeography, 17, 236–245. https://doi.org/10.1111/j.1466-8238.2007.00360.x

[ece33666-bib-0025] Scholes, R. J. , & Archer, S. R. (1997). Tree‐grass interactions in savannas. Annual Review of Ecology and Systematics, 28, 517–544. https://doi.org/10.1146/annurev.ecolsys.28.1.517

[ece33666-bib-0026] Sitters, J. , Edwards, P. J. , & Olde Venterink, H. (2013). Increases of soil C, N, and P pools along an acacia tree density gradient and their effects on trees and grasses. Ecosystems, 16, 347–357. https://doi.org/10.1007/s10021-012-9621-4

[ece33666-bib-0027] Sitters, J. , Maechler, M. J. , Edwards, P. J. , Suter, W. , & Olde Venterink, H. (2014). Interactions between C:N: P stoichiometry and soil macrofauna control dung decomposition of savanna herbivores. Functional Ecology, 28, 776–786. https://doi.org/10.1111/1365-2435.12213

[ece33666-bib-0028] Staver, A. C. , Bond, W. J. , Stock, W. D. , van Rensburg, S. J. , & Waldram, M. S. (2009). Browsing and fire interact to suppress tree density in an African savanna. Ecological Applications, 19, 1909–1919. https://doi.org/10.1890/08-1907.1 19831079 10.1890/08-1907.1

[ece33666-bib-0029] Tjoelker, M. G. , Craine, J. M. , Wedin, D. , Reich, P. B. , & Tilman, D. (2005). Linking leaf and root trait syndromes among 39 grassland and savannah species. New Phytologist, 167, 493–508. https://doi.org/10.1111/j.1469-8137.2005.01428.x 15998401 10.1111/j.1469-8137.2005.01428.x

[ece33666-bib-0030] van der Waal, C. , Kool, A. , Meijer, S. S. , Kohi, E. , Heitkönig, I. M. A. , de Boer, W. F. , … de Kroon, H. (2011). Large herbivores may alter vegetation structure of semi‐arid savannas through soil nutrient mediation. Oecologia, 165, 1095–1107. https://doi.org/10.1007/s00442-010-1899-3 21225433 10.1007/s00442-010-1899-3 PMC3057003

[ece33666-bib-0031] van Langevelde, F. , van de Vijver, C. , Kumar, L. , van de Koppel, J. , de Ridder, N. , van Andel, J. , … Rietkerk, M. (2003). Effects of fire and herbivory on the stability of savanna ecosystems. Ecology, 84, 337–350. https://doi.org/10.1890/0012-9658(2003)084[0337:EOFAHO]2.0.CO;2

[ece33666-bib-0032] Vitousek, P. M. , Cassman, K. , Cleveland, C. , Crews, T. , Field, C. B. , Grimm, N. B. , … Sprent, J. I. (2002). Towards an ecological understanding of biological nitrogen fixation. Biogeochemistry, 57, 1–45. https://doi.org/10.1023/A:1015798428743

